# High-Risk Sexual Behavior Is Associated with Postexposure Prophylaxis Nonadherence among Men Who Have Sex with Men Enrolled in a Combination Prevention Intervention

**DOI:** 10.1155/2013/210403

**Published:** 2013-07-10

**Authors:** Jesse B. Fletcher, Joshua A. Rusow, Hung Le, Raphael J. Landovitz, Cathy J. Reback

**Affiliations:** ^1^Friends Research Institute, Inc., 1419 N. La Brea Avenue, Los Angeles, CA 90028, USA; ^2^UCLA Center for Clinical AIDS Research & Education, David Geffen School of Medicine, University of California, Los Angeles, CA 90095, USA; ^3^UCLA Integrated Substance Abuse Programs, Semel Institute for Neuroscience and Human Behavior, University of California at Los Angeles, CA 90025, USA

## Abstract

Methamphetamine use among men who have sex with men (MSM) is associated with increased HIV prevalence, due to increased engagement in high-risk sexual behavior. Fifty-three HIV-negative, methamphetamine-using MSM were enrolled in a biobehavioral combination prevention intervention in Los Angeles, CA, to assess the feasibility of administering postexposure prophylaxis (PEP) in combination with contingency management (CM) to prevent HIV seroconversion. The study combined a CM behavioral intervention targeting reductions in methamphetamine use with a PEP biomedical intervention for HIV prevention. Those who reported recent exposure to HIV were initiated on tenofovir/emtricitabine- (Truvada-) based PEP (*n* = 35). This secondary analysis sought to determine whether sexual risk taking was associated with PEP adherence. Regression analyses controlling for participant sociodemographics demonstrated that, at baseline, increased number of lifetime sexually transmitted diseases (STDs; Coef. = −0.07; 95% CI= (−0.12) – (−0.01)) and recent episodes of unprotected anal intercourse (UAI; Coef. = −0.01; 95% CI= (−.01) – (−0.002)) were associated with reductions in medication adherence. Given these associations between baseline sexual risk and PEP adherence, providers working with high-risk MSM may look to target reductions in sexual risk taking; this will reduce direct risk of HIV infection and may work to optimize medication adherence in the case of PEP initiation.

## 1. Introduction

In the United States, men who have sex with men (MSM) exhibit disproportionately high incidence and prevalence of HIV infection. MSM represent an estimated 4.7–9.2% of the total United States (US) population [1] yet, in 2009, accounted for 61% of all new HIV infections in the US [2]. Risk factors for HIV infection have been widely documented and among MSM in the US include unprotected anal intercourse, high number of male partners [3], and methamphetamine use [4, 5]. Postexposure prophylaxis (PEP) is a biomedical intervention intended to reduce the likelihood of HIV seroconversion after exposure to the virus. Combination prevention approaches combine biomedical interventions like PEP with behavioral (e.g., cognitive behavioral therapy, motivational interviewing) and/or structural (e.g., needle exchange, condom distribution) interventions, thereby optimizing the likelihood of proper adherence to the PEP medication while also reducing the risk factors placing participants at risk for HIV seroconversion.

### 1.1. Combination Prevention Interventions

Combination prevention interventions integrate behavioral, biomedical, and/or structural intervention strategies in the attempt to maximize the likelihood of intervention success and optimize intervention outcomes [6]. The increasing application of combination prevention interventions in the US represents the impact of the National HIV/AIDS Strategy (NHAS) [7], which highlighted the importance of combination approaches in successfully reducing HIV transmission. Recent disease epidemic models have demonstrated that movement away from purely behavioral interventions towards combination efforts may reduce the long-term costs of care as well as the overall number of persons living with HIV in the US [8, 9]. In addition, such efforts may be most cost effective when targeted for communities or populations at highest risk for HIV transmission.

### 1.2. Methamphetamine Use and HIV Sexual Risk among MSM

Methamphetamine is the most frequently used substance among MSM, following alcohol and marijuana, particularly in urban centers along the western US [10]. Among MSM, methamphetamine use has been associated with increased high-risk sexual behaviors [11–14]. Results have shown an ecological association between intensity of methamphetamine use and HIV infection; as the intensity of methamphetamine use increases, so does the likelihood of an observed HIV-positive status [5]. 

### 1.3. Postexposure Prophylaxis

Postexposure prophylaxis is the preventative strategy of taking 28 days of antiretroviral therapy (ART), initiated rapidly after an exposure to HIV, to reduce the odds of HIV acquisition [15]. Though there is currently limited clinical data for nonoccupational use [16], PEP is currently recommended for use after high-risk sexual exposures and/or needle sharing [15]. Failure to properly adhere to a prescribed PEP treatment regimen may not only fail to deter HIV seroconversion, but may also result in viral resistance which would make subsequent treatment of the disease with ART more difficult [16]. As such, difficult to treat populations (e.g., substance users, the homeless) may require additional intervention support in order to ensure proper adherence to a prescribed PEP regimen.

### 1.4. The Efficacy and Feasibility of PEP for High-Risk MSM

In a randomized noninferiority trial conducted in San Francisco [17], PEP-related adherence outcomes were contrasted across groups of low- and high-sexual risk-taking MSM. Though noninferiority was corroborated in the group of low risk-taking MSM, the high-risk MSM required more counseling support to achieve comparable results. Those that did not receive enhanced counseling displayed marginally lower rates of PEP course completion; evidence from animal models suggests that truncated courses of PEP severely compromise the efficacy of the intervention [18]. 

Sexual risk-taking MSM prescribed PEP may thus require additional motivation and support, such as that provided by combination prevention methods, to optimize levels of medication adherence and to maximize likelihood of course completion. Preliminary pilot data from a behavioral/biomedical combination prevention intervention in Los Angeles demonstrated that high-risk, methamphetamine-using MSM receiving PEP and undergoing a contingency management intervention to reduce methamphetamine use produced levels of medication adherence and rates of course completion comparable to historical cohorts [19–21]. The behavioral component of the intervention (i.e., contingency management to reduce methamphetamine use) was associated with reductions in participant methamphetamine use; it was also demonstrated that years of heavy methamphetamine use at baseline, and ongoing methamphetamine use during the course of the study were both associated with suboptimal PEP medication adherence and/or course completion. 

**Figure 1:**
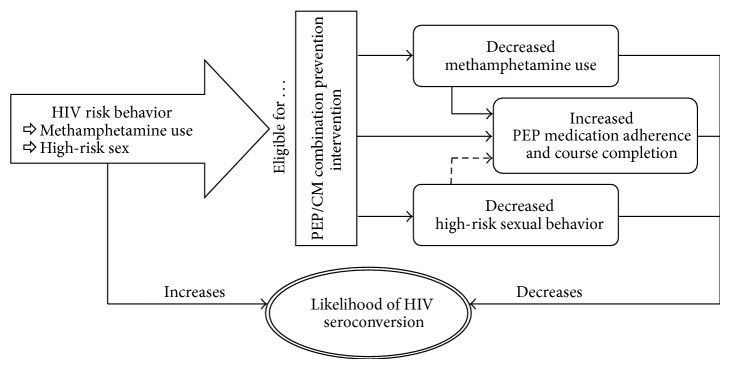
Proposed causation model.

 MSM with multiple risk factors for HIV infection, such as high-risk sexual behaviors and methamphetamine use, may represent a prime target population for combination prevention interventions designed to simultaneously reduce HIV risk behavior and incident HIV infection. A causation model illustrating the proposed linkages between methamphetamine use, high-risk sexual behaviors, the PEP/CM combination prevention intervention, and likelihood of HIV seroconversion is provided in Figure 1. This secondary analysis sought to determine if lifetime and/or recent levels of high-risk sexual behavior were associated with poorer medication adherence and/or course completion among a sample of high-risk HIV negative, methamphetamine-using MSM. It was hypothesized that both recent and lifetime sexual risk taking would be associated with reductions in PEP medication adherence and odds of course completion. This hypothesized association would corroborate the dashed arrow in Figure 1.

## 2. Materials and Methods

The Institutional Review Boards for UCLA and Friends Research Institute provided oversight for all study activities and approved all study-related documents, materials, and procedures. Exact procedures of the contingency management behavioral intervention (including payout schedules) are published elsewhere [21]. 

### 2.1. Participants

Participants were recruited between March 2009 and August 2010 using targeted ads posted in local gay magazines and the distribution of flyers and club cards in the settings where methamphetamine-using MSM congregate (e.g., dance clubs, bathhouses, coffee houses, and gyms). Potential participants were eligible if they self-identified as MSM, were at least 18 years of age, HIV uninfected on rapid HIV ELISA testing, self-reported methamphetamine use within the previous 30 days, and reported unprotected anal intercourse (UAI) with an HIV-positive or HIV-serostatus-unknown partner in the previous 90 days. 

### 2.2. Study Procedures

All study procedures were conducted at a community research site in Los Angeles, CA. At a baseline visit, all eligible participants underwent informed consent, completed baseline assessments, received rapid HIV testing (OraQuick Advance, OraSure technologies, Bethlehem, PA), provided specimens for syphilis, Neisseria gonorrhoeae, and Chlamydia testing, and received a medical examination. Those who reported a high-risk sexual or drug exposure episode with an HIV-positive or serostatus-unknown source within the preceding 72 hours immediately initiated tenofovir isoproxil fumarate + emtricitabine (Truvada, Gilead Sciences), one tablet daily, for 28 days. All other participants received a 4-day “starter pack” of Truvada to be initiated only in the future case of a high-risk exposure to HIV. Thirty-five participants initiated PEP during the study period and comprise the analytic sample. One incident of HIV seroconversion was observed in a participant who reported medication nonadherence and multiple subsequent sexual exposures. Further study procedures and information on the incident seroconversion are described in detail elsewhere [21]. 

### 2.3. Assessments

Baseline assessments included demographics, methamphetamine use (DSM-IV-TR), sexual risk behaviors (Behavioral Questionnaire-Amphetamine [BQA-II]), medication adherence, HIV serostatus, and sexually transmitted diseases (STDs, urine sample, self-performed rectal swab for nucleic acid amplification [NAAT] for *N. gonorrhoeae* and C. trachomatis and pharyngeal swab for *N. gonorrhoeae*, and syphilis testing via serum rapid plasma reagin [RPR] assay). HIV and STD testing were performed at three-month followup; HIV RNA testing was performed only in the event of suspicion of acute HIV seroconversion. Further specifics regarding biologic testing and monitoring are provided elsewhere [21]. 

To determine medication adherence, participants were asked to report if they had missed any doses of the Truvada medication each time they came on site for a scheduled study appointment or to pick up additional medication. Proportional PEP adherence is defined as the number of doses taken divided by the total number of doses prescribed (e.g., X/28). If a participant missed more than 3 consecutive doses at any point during the 28-day regimen, they were discontinued from the medication and were considered to have not achieved course completion. Thus, course completion was a dichotomous (0/1) variable that indicates that a participant never missed more than three consecutive doses and was thus able to continue taking the prescribed medication through to the last dose.

There were two measures of high-risk sexual behaviors in this study. Recent high-risk sexual behavior was operationalized as the self-reported number of episodes of UAI during past six months at baseline. Lifetime high-risk sexual behavior was operationalized as the self-reported number of STDs acquired over the life course at baseline. 

### 2.4. Statistical Analysis

For descriptive analyses, counts and percentages were provided for nominal variables, while means and standard deviations were provided for continuous or count variables. Multivariate analyses included both ordinary least squares (OLS) regressions (for analyses of proportional medication adherence) and logistic regressions (for analyses of course completion; 0 = did not complete course, 1 = completed course). All multivariate analyses included participants' race/ethnicity and sexual identity as statistical controls. In no case were participants' race/ethnicity or sexual identity significantly associated with PEP-related outcomes, and thus their coefficient estimates were omitted. Three participants were unwilling to disclose sexual risk behaviors at baseline. Due to the small sample sizes, results are reported as significant beginning at *P* ≤ 0.1. All analyses were carried out using Stata version 10SE (StataCorp, College Station, TX).

## 3. Results

Participant sociodemographics are presented in Table 1. Most participants (60%) identified as white, with most nonwhite participants identifying as Hispanic/Latino (*n* = 9; 25.7%). Most participants identified as gay (85.7%), and most reported having a high school diploma or GED equivalent (60.0%). Self-reported annual income in the sample was low, with nearly half (48.6%) of the sample reporting yearly earnings of less than or equal to $15,000, and nearly three-quarters of the sample (74.3%) reporting earning less than or equal to $30,000 a year. Most participants reported renting or owning a house/apartment (54.3%), though a sizable minority reported being homeless (11.4%). Self-reported lifetime history of STDs was common (*M*_STD_ = 1.8; SD = 2.2), as were counts of recent episodes of UAI (*M*_UAI_ = 11.9; SD = 26.5).

Table 2 presents overall proportional PEP adherence rates; nearly half (48.6%) of all participants who initiated PEP took all 28 doses of the Truvada medication. Another five (14.3%) participants were at least 90% adherent to the medication regimen, meaning 62.9% of the PEP-initiators were ≥90% adherent. Eight PEP-initiators (22.9%) failed to complete half of the 28-day treatment regimen. Of the 35 PEP initiators, 25 (71.4%) continued taking the medication with sufficient frequency to avoid discontinuation and completed the prescribed course (i.e., course completion). 

Table 3 provides the results of six separate multivariate regressions, three models for each of the two PEP-related outcomes (medication adherence, course completion). Results included under Model 1 provide associations between participants' lifetime number of STDs and medication adherence/course completion. When controlling for covariates, participants' lifetime number of STDs was significantly associated with both PEP adherence and course completion. For each additional STD reported at baseline, participants' estimated proportional medication adherence reduced by 0.07 (approximately 2 of the 28 total doses), and their odds of course completion reduced by an estimated 31%. 

Results appearing under Model 2 provide associations between recent episodes of UAI and medication adherence/course completion. Participants' self-reported count of recent UAI was significantly associated with both participants' PEP adherence and odds of course completion. For each additional episode of UAI in the past six months, participants' estimated proportional medication adherence reduced by 0.01 and their odds of completing the PEP course reduced by an estimated 6%. Analyses appearing under Model 3 regress PEP outcomes on STDs and episodes of UAI simultaneously. Results revealed that only the number of recent episodes of UAI remains significantly associated with PEP outcomes when all cofactors are included simultaneously. However, an examination of the coefficients of determination (i.e., *R* ^2^, Pseudo-*R* ^2^) revealed a nontrivial increase in the amount of variance explained in each model when including both factors simultaneously. This indicates that the significance test on the coefficient for STDs is likely being influenced by the small sample size (*n* = 32) and relatively large number of factors (*k* = 4) included in the model and may represent a type II hypothesis testing error rather than a genuine lack of statistical association. Additional factors tested for inclusion in the analysis included self-reported methamphetamine use (or methamphetamine and an other substance use) during sex, current relationship status, and total number of recent nonprimary sexual partners. In no case were these factors significantly associated with PEP adherence, and were thus omitted from the final models.

## 4. Discussion

Given the associations between methamphetamine use, high-risk sexual behaviors, and HIV infection/transmission among MSM, there is a need to develop effective interventions to prevent the acquisition and transmission of HIV among this extremely high-risk population [14]. At baseline, participants reported an average of almost two prior STDs and 12 recent episodes of UAI in the past six months. This was commensurate with the eligibility criteria of the study, one of which was self-report UAI (either receptive or insertive) with an HIV-positive or serostatus-unknown partner in the past three months. Given the well-documented association between methamphetamine use and high-risk sexual behaviors among MSM, as well as the urgency to target sexual risk behaviors among MSM in HIV prevention efforts, it is important to determine whether sexual risk taking impacted medication adherence rates and/or the likelihood of course completion among this sample of high-risk, methamphetamine-using MSM.

Findings demonstrated that medication adherence was comparable with other, nonsubstance using populations [19, 20, 22], with 63% of all participants who initiated PEP achieving a minimum of 90% adherence to the 28-day medication regimen. When controlling for participant race/ethnicity and sexual identity, separate analytical models revealed that increased numbers of lifetime STDs and recent episodes of UAI were both associated with reductions in PEP medication adherence and course completion. When both factors (and the aforementioned controls) were estimated simultaneously, only recent episodes of UAI remained significantly associated with the PEP-related outcomes, though the likelihood of a type II hypothesis testing error for the coefficient on STDs is high. When all factors were included simultaneously, analytic models succeeded at explaining a third of all variance in medication adherence and a quarter of all variation in course completion.

Individuals undertaking high levels of sexual risk are prime candidates for efficacious HIV prevention strategies, including administration of biomedical interventions such as PEP. However, insofar as these same individuals are empirically less likely to properly adhere to such interventions, there is potential for the development of drug-resistant strains of HIV or other risks associated with suboptimal medication adherence. As such, the acceptability of PEP among populations unlikely to adhere to prescribed regimens may be drawn sharply into question. Any evidence revealing associations between past behavior and estimated PEP-adherence must, then, be closely attended.

The intention of combination prevention interventions is to provide, through the integration of multimodal intervention techniques, additional support and motivation to those at highest risk for intervention noncompliance to complete their assigned medication regimen and maximize the likelihood of HIV nonseroconversion. The PEP/CM combination prevention intervention described here was designed in part to reduce methamphetamine use among participants; prior results indicated that the intervention was associated with reductions in methamphetamine use and high-risk sexual behavior [21]. Reduction of high-risk sexual behaviors was not a direct targeted outcome of the CM behavioral intervention, though previous research has also demonstrated that reductions in methamphetamine use among MSM were accompanied by reductions in high-risk sexual behaviors [23]. Given the results presented here, combination prevention interventions that provide PEP to high-risk MSM should consider the inclusion of behavioral interventions explicitly designed to reduce substance use and concomitant high-risk sexual behaviors. In this way, sexual risk taking may be preemptively targeted for reduction, increasing the likelihood that a PEP regimen will be adhered to and/or completed. 

Such reductions in high-risk sexual behaviors would benefit high-risk MSM in multiple ways, including reducing the need for PEP, lowering risk of infection with HIV and STDs, and maximizing adherence and likelihood of course completion if PEP is initiated. The direct reductions in the need for PEP initiation as well as decreased risk for infection with HIV or other STDs are of primary interest. However, for those who reduce but do not eliminate high-risk behaviors and still require PEP initiation, results presented here also indicate that PEP-related outcomes may be maximized, further decreasing the likelihood of HIV seroconversion. Perhaps most promisingly, given the intersecting and reinforcing nature of methamphetamine use and high-risk sexual behaviors among MSM, combination prevention interventions designed to reduce both while simultaneously providing biomedical interventions to avoid seroconversion would provide a more holistic, syndemic approach to HIV-prevention among high-risk MSM. 

This study was limited by the face-to-face, self-reported nature of the sexual risk data collected at baseline. Furthermore, given the highly specialized nature of the sample (methamphetamine-using MSM living in Los Angeles county engaged in at least one high-risk sexual behavior in the past three months), results presented here may not be generalizable to other populations. Lastly, the small sample size and use of multivariate inferential statistics necessitated the use of relaxed statistical reporting standards, increasing the risk of both type I and type II hypothesis testing errors. However, in spite of these limitations, the results presented here provide evidence that both recent and lifetime high-risk sexual behaviors are associated with PEP-related outcomes. Given that high-risk, methamphetamine-using MSM are often targeted for HIV-prevention interventions, including PEP combination prevention interventions, the results presented here provide important evidence to researchers looking to develop combination prevention interventions to this and similar high-risk populations. 

High-risk sexual behavior is a serious public health concern among MSM communities across the US. As indicated by the recently implemented NHAS, combination prevention interventions are being tested for their efficacy in augmenting purely biomedical means of preventing HIV transmission in this and other populations disproportionately affected by the HIV epidemic. The ability to effectively determine factors endemic to such high-risk populations that may prevent proper implementation and adherence to prescribed biomedical interventions is crucial to this effort. The results presented here indicate that self-reported sexual risk taking is associated with reduced rates of medication adherence and likelihood of course completion among methamphetamine-using MSM. Thus, future combination prevention interventions targeting high-risk MSM should include behavioral intervention components specifically designed to reduce high-risk sexual behaviors that can, thereby, serve to optimize biobehavioral outcomes. 

## Figures and Tables

**Table 1:** PEP-initiator sociodemographic characteristics (*N* = 35).

Characteristic	*N* (%) or mean (SD)
Age	34.1 (7.4)
Race/ethnicity	
Caucasian/white	21 (60.0%)
Non-White	14 (40%)
Sexual identity	
Gay	30 (85.7%)
Non-gay	5 (14.3%)
Educational attainment	
Less than HS	1 (2.9%)
HS Diploma/GED	21 (60.0%)
BA/BS	9 (25.7%)
Post graduate	4 (11.4%)
Annual income	
≤$15,000	17 (48.6%)
$15,001–$30,000	9 (25.7%)
$30,001–$60,000	6 (17.1%)
>$60,000	3 (8.6%)
Housing status	
Own/Rent House/Apt.	19 (54.3%)
Group Housing/Sober Living	3 (8.6%)
With Family/Friends	9 (25.7%)
Homeless	4 (11.4%)
Sexually transmitted diseases	
Lifetime	1.8 (2.2)
# Times unprotected anal intercourse^a^	
Past 6 months	11.9 (26.5)

^a^ *n* = 32.

**Table 2:** Adherence to post-exposure prophylaxis medication regimen.

Proportional adherence	Freq.	Percent	Cumulative
0.04	3	8.6	8.6
0.07	2	5.7	14.3
0.14	1	2.9	17.1
0.25	1	2.9	20.0
0.46	1	2.9	22.9
0.57	2	5.7	28.6
0.77	1	2.9	31.4
0.82	1	2.9	34.3
0.89	1	2.9	37.1
0.93	1	2.9	40.0
0.96	4	11.4	51.4
1.00	17	48.6	100.0

Total	35	100	

**Table 3:** Multivariate analyses of PEP adherence and course completion.

Outcome variable	Factor(s)	Model 1 (*N* = 35)		Model 2 (*n* = 32)		Model 3 (*n* = 32)	
		Coef.	95% CI	Coef.	95% CI	Coef.	95% CI

PEP adherence	STDs	−0.07∗	(−0.12)–(−0.01)	—	—	−0.04	(−0.10)–0.03
	UAI	—	—	−0.01∗∗	(−0.01)–(−0.002)	−0.01∗∗	(−0.01)–(−0.002)
		*R* ^2^ = 0.17		*R* ^2^ = 0.28		*R* ^2^ = 0.32	

		AOR	95% CI	AOR	95% CI	AOR	95% CI

Course completion	STDs	0.69^†^	0.46–1.01	—	—	0.71	0.42–1.21
	UAI	—	—	0.94°	0.87–1.01	0.94°	0.87–1.01
		Pseudo *R* ^2^ = 0.12		Pseudo *R* ^2^ = 0.22		Pseudo *R* ^2^ = 0.27	

°*P* ≤ 0.1; ^†^ *P* = 0.058; ∗*P* ≤ 0.05; ∗∗*P* ≤ 0.01.

Controls: Race/Ethnicity, Sexual Identity.

STDs: Sexually Transmitted Diseases (Lifetime).

UAI: Unprotected Anal Intercourse (past 6 months).
